# Supplementary figures and images for: Antibacterial and anti-adherence effects of a plant extract mixture (PEM) and its individual constituent extracts (Psidium sp., Mangifera sp., and Mentha sp.) on single- and dual-species biofilms
Source: PeerJ. 2016 Oct 5;4:e2519. doi: 10.7717/peerj.2519 (PMC5068394; doi:10.7717/peerj.2519)

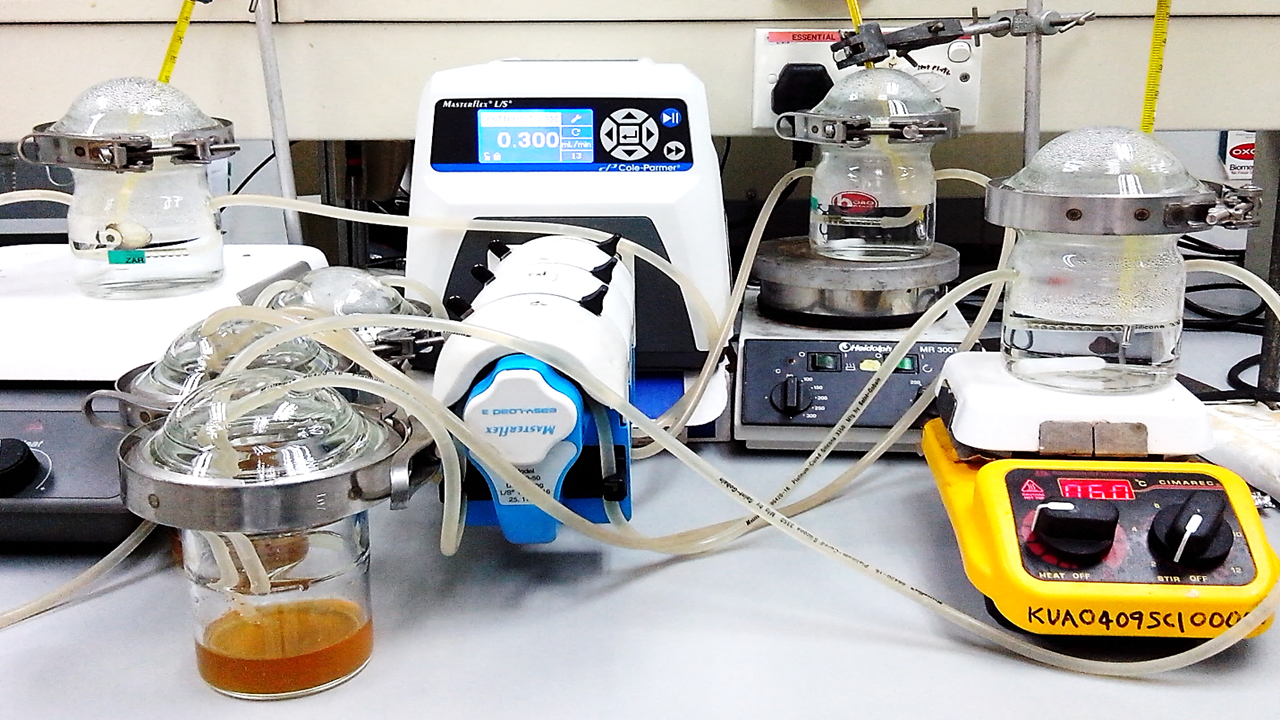

Supplement: Figure S1 [file peerj-04-2519-s001.png]

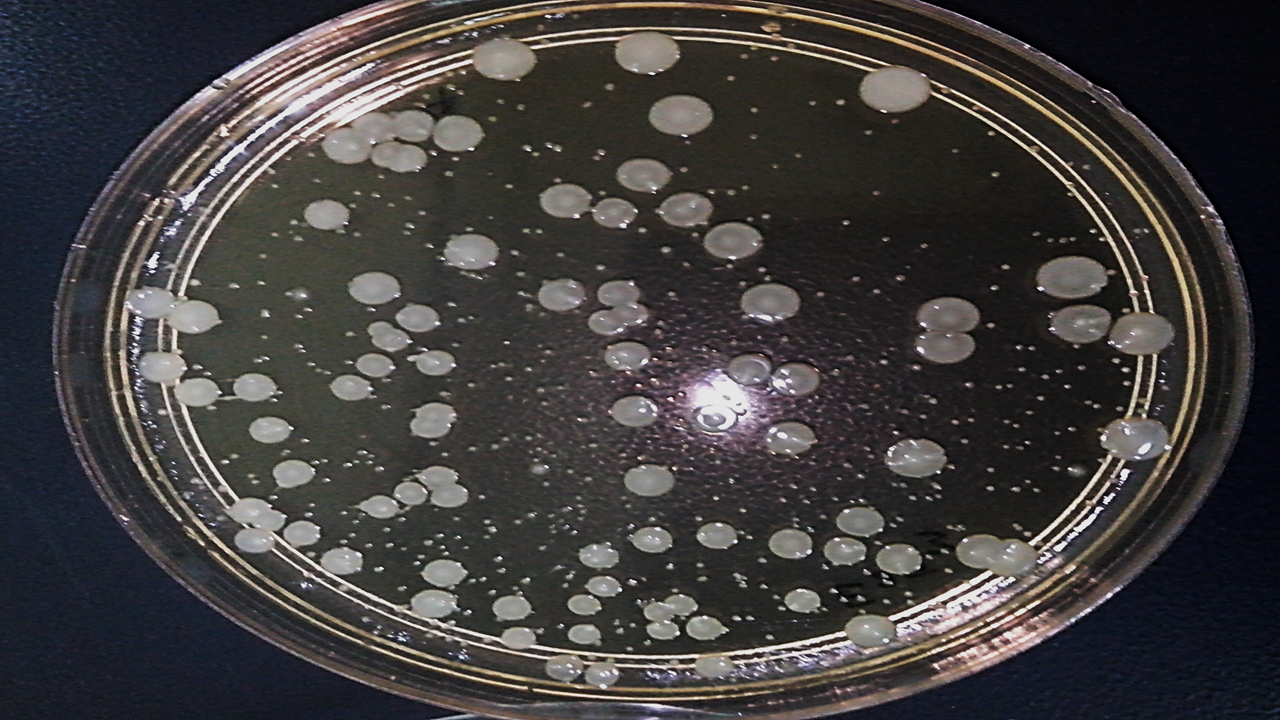

Supplement: Figure S2 [file peerj-04-2519-s002.png]
